# Supplementary figures and images for: Evaluating the impact of sex bias on AI models in musculoskeletal ultrasound of joint recess distension
Source: PLoS One. 2025 Nov 12;20(11):e0332716. doi: 10.1371/journal.pone.0332716 (PMC12611148; doi:10.1371/journal.pone.0332716)

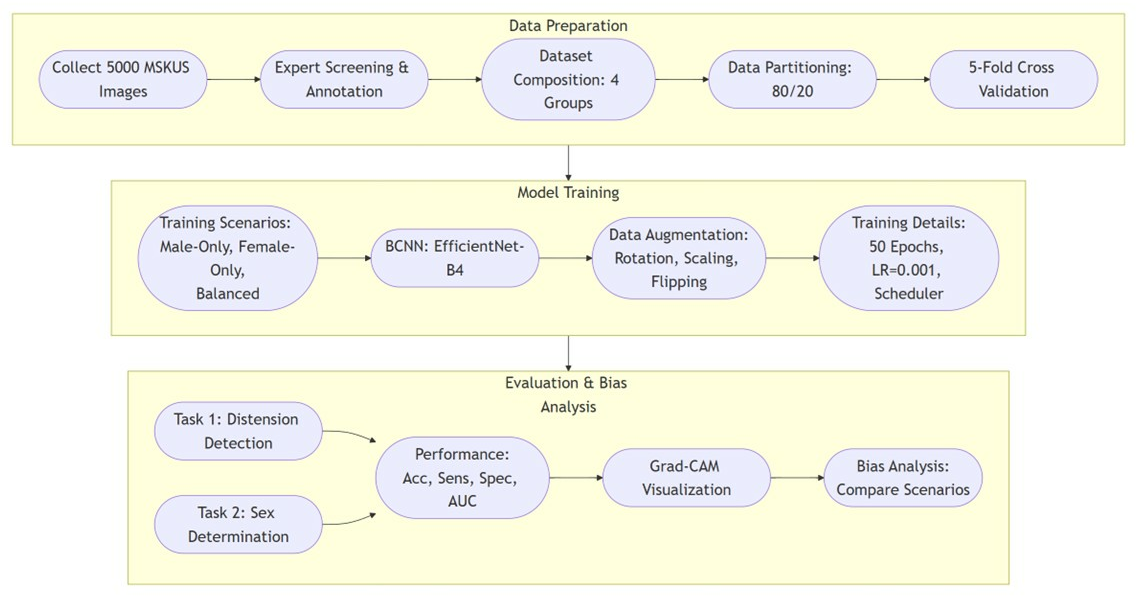

Supplement: S1 Fig — The pipeline consists of three main stages: (1) Data Preparation, including expert screening, dataset composition, and partitioning; (2) Model Training, utilizing EfficientNet-B4 with different training scenarios and data augmentation strategies; and (3) Evaluation and Bias Analysis, which involves performance assessment, Grad-CAM visualization, and bias comparison across training conditions. (TIF) [file pone.0332716.s001.tif]

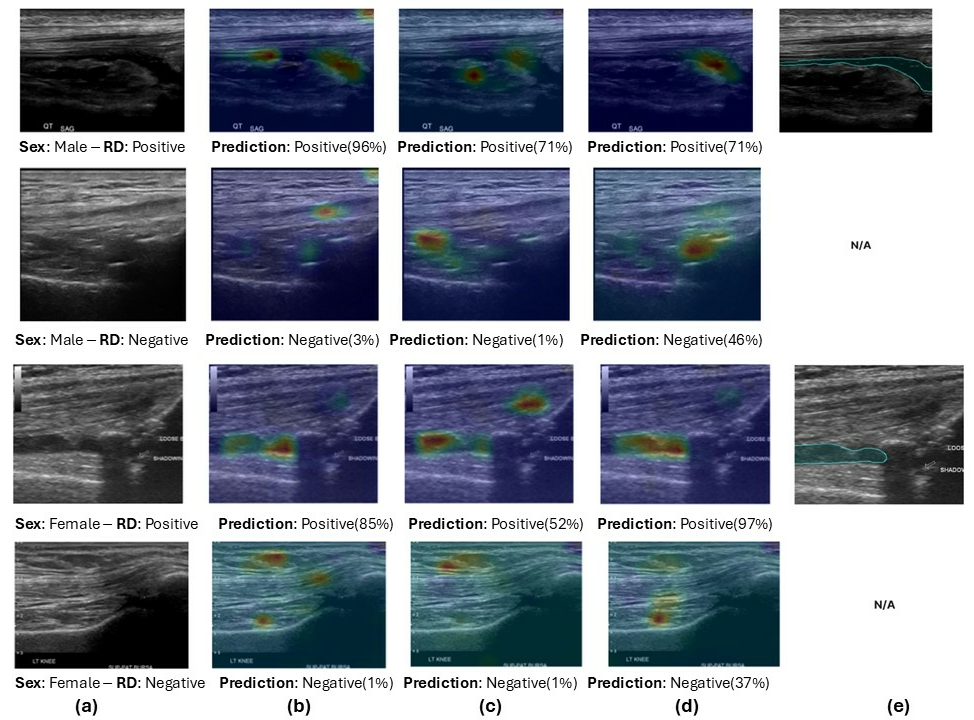

Supplement: S2 Fig — Column (a) displays the original captured ultrasound image; column (b) shows the heatmap for models trained on male data; column (c) for models trained on female data; and column (d) for models trained on a combined dataset. Column (e) includes a segmentation mask of synovial recess distension as a reference. Model predictions are presented beneath each heatmap. Note that while model predictions align, the focus areas vary depending on the sex composition of the training data. This visualization aids in understanding how training variability impacts model precision and bias. RD: Synovial recess distension. (TIF) [file pone.0332716.s002.tif]

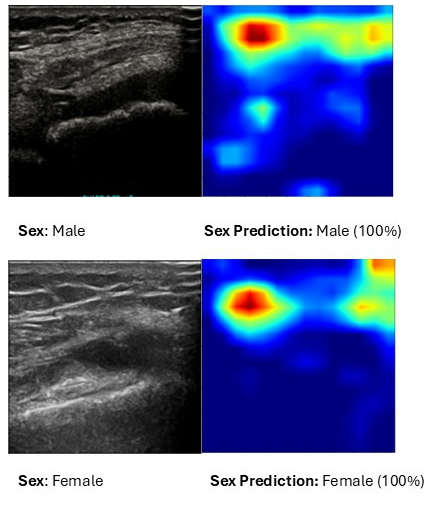

Supplement: S3 Fig — The heatmaps highlight the regions that contribute most to the model’s predictions, illustrating potential sex-specific imaging features. These visualizations provide insights into the model’s decision-making process and help assess possible sources of bias in classification. (TIF) [file pone.0332716.s003.tif]
