# Supplementary material for: Evaluating the impact of sex bias on AI models in musculoskeletal ultrasound of joint recess distension
Source: PLoS One. 2025 Nov 12;20(11):e0332716. doi: 10.1371/journal.pone.0332716 (PMC12611148; doi:10.1371/journal.pone.0332716)
Supplement: S1 Table — This table presents accuracy, sensitivity, specificity, and AUC metrics for models trained to detect knee synovial recess distension on male-only, female-only, and combined ultrasound datasets, tested across a sex-balanced dataset. (DOCX) [file pone.0332716.s004.docx]

|  | **Accuracy** | **Sensitivity** | **Specificity** | **AUC** |
| --- | --- | --- | --- | --- |
| **Trained on Males** | 83.98% | 83.11% | **87.86%** | 0.9225 |
| **Trained on Females** | 87.47% | 88.46% | 83.1% | **0.9269** |
| **Trained on Both** | **87.73%** | **88.51%** | 84.29% | 0.9209 |
