# Supplementary material for: Evaluating the impact of sex bias on AI models in musculoskeletal ultrasound of joint recess distension
Source: PLoS One. 2025 Nov 12;20(11):e0332716. doi: 10.1371/journal.pone.0332716 (PMC12611148; doi:10.1371/journal.pone.0332716)
Supplement: S2 Table — The table quantifies accuracy, sensitivity, specificity, and AUC for knee synovial recess distension detection in male and female patients. M: Male. F: Female. Sp: Subpopulation in the test set. (DOCX) [file pone.0332716.s005.docx]

|  | **Accuracy** | | | **Sensitivity** | | | **Specificity** | | | **AUC** | |
| --- | --- | --- | --- | --- | --- | --- | --- | --- | --- | --- | --- |
|  | M. Sp. | F. Sp. | M. Sp. | | F. Sp. | M. Sp. | | F. Sp. | M. Sp. | | F. Sp. |
| **Trained on Males** | 84.5% | 83.3% | 84.26% | | 81.75% | **85.64%** | | **89.78%** | **0.8495** | | 0.8576 |
| **Trained on Females** | 87.42% | **87.87%** | **89.61%** | | 87.73% | 76.92% | | 88.44% | 0.8327 | | **0.8809** |
| **Trained on Both** | **87.51%** | **87.87%** | 89.08% | | **87.83%** | 80% | | 88% | 0.8454 | | 0.8792 |
