# Supplementary material for: Evaluating the impact of sex bias on AI models in musculoskeletal ultrasound of joint recess distension
Source: PLoS One. 2025 Nov 12;20(11):e0332716. doi: 10.1371/journal.pone.0332716 (PMC12611148; doi:10.1371/journal.pone.0332716)
Supplement: S3 Table — Sp: Subpopulation in the test set. w/: with. w/o: without. RD: knee synovial recess distension. (DOCX) [file pone.0332716.s006.docx]

|  | **Male Sp w/ RD** | **Male Sp w/o RD** | **Female Sp w/ RD** | **Female Sp w/o RD** |
| --- | --- | --- | --- | --- |
| **Trained on Males** | 84.26% | **85.64%** | 81.75% | **89.78%** |
| **Trained on Females** | **89.61%** | 76.92% | 87.73% | 88.44% |
| **Trained on Both** | 89.08% | 80% | **87.83%** | 88% |
