# Supplementary material for: Evaluating the impact of sex bias on AI models in musculoskeletal ultrasound of joint recess distension
Source: PLoS One. 2025 Nov 12;20(11):e0332716. doi: 10.1371/journal.pone.0332716 (PMC12611148; doi:10.1371/journal.pone.0332716)
Supplement: S4 Table — This table displays the outcomes of a 5-fold cross-validation for the machine learning knee synovial recess distension model, trained with datasets characterized by specific sex distributions (male, female, or a combination of both). The validation sets are derived directly from the respective training populations, thereby mirroring the sex distribution of each training set. (DOCX) [file pone.0332716.s007.docx]

|  | **Accuracy** | **Sensitivity** | **Specificity** | **AUC** |
| --- | --- | --- | --- | --- |
| **Trained on Males** | 83.3%±0.01% | 83.69%±0.03% | 83.07%±0.03% | 89.58%±0.02% |
| **Trained on Females** | **87.65%±0.04%** | **88.95%±0.04%** | **86.54%±0.05%** | **93.68%±0.04%** |
| **Trained on Both** | 86.65%±0.02% | 87.76%±0.03% | 85.61%±0.04% | 92.69%±0.01% |
