# Supplementary material for: Evaluating the impact of sex bias on AI models in musculoskeletal ultrasound of joint recess distension
Source: PLoS One. 2025 Nov 12;20(11):e0332716. doi: 10.1371/journal.pone.0332716 (PMC12611148; doi:10.1371/journal.pone.0332716)
Supplement: Attachment — Submitted filename: Response to Reviewers.docx [file pone.0332716.s009.docx]

We thank all the reviewers for their constructive comments, and the Associate Editor for handling the review of this paper. Please find the point-by-point responses to all the comments in blue font. The modifications to the original manuscript are also marked in blue font. Please note that due to adding new figures, the figure numbers have been updated in the revised manuscript.

Review Comments to the Author

Please use the space provided to explain your answers to the questions above. You may also include additional comments for the author, including concerns about dual publication, research ethics, or publication ethics. (Please upload your review as an attachment if it exceeds 20,000 characters)

Reviewer #1: In this study, the authors assessed the impact of sex bias on a specific internal test set using CNNs for diagnosing knee joint recess distension using ultrasound imaging.

My specific comments are as follows:

COMMENTS:

Comment 1: “The integration of artificial intelligence (AI), particularly convolutional neural networks (CNNs), in medical imaging is revolutionizing early diagnosis practices (8–10).”

This is wrong. Because “particularly” CNNs are not the state of the art anymore. The vision transformers which are the backbones of foundation models are the state of the art.

**Response:** We appreciate the reviewer’s suggestion regarding Vision Transformers (ViTs) as a state-of-the-art approach in medical imaging. However, the primary objective of this study is not to benchmark different deep learning architectures but rather to explore how demographic biases in training data affect the performance of AI models in musculoskeletal ultrasound. Given that demographic biases arise from imbalances in data representation rather than specific model architectures, the effect of bias on ViTs is expected to be similar to that observed in CNN-based models.

We selected CNN-based models, specifically EfficientNet-B4, due to their efficiency, widespread adoption in medical imaging applications, and suitability for datasets of moderate size. While ViTs have demonstrated superior performance in large-scale computer vision tasks, their application in clinical AI is still evolving, and they typically require significantly larger datasets and computational resources to outperform CNNs.

Nevertheless, we acknowledge the potential of ViTs in future medical AI applications. To reflect this, we have added the following paragraphs in the INTRODUCTION Section lines 127-133:

“While ViTs introduce self-attention mechanisms that enhance feature extraction, the effect of dataset bias on ViTs is expected to be similar to that on CNNs—model fairness and generalization are primarily dictated by the composition and diversity of the training data, rather than architecture choice. CNNs, particularly architectures like EfficientNet, remain widely used in real-world medical settings because of their computational efficiency, established performance, and ability to generalize well on moderate-sized datasets (25,26).”

and DISCUSSION Section, Sub-Section 4-1, lines 491-496:

“Bias in AI models is primarily driven by dataset composition rather than architecture choice. Regardless of whether CNNs or ViTs are used, training data diversity and representativeness remain the most critical factors in ensuring fairness and mitigating bias in diagnostic models. Future research could investigate whether ViTs amplify or mitigate demographic biases in musculoskeletal ultrasound classification by leveraging their attention-based feature extraction.”

25.Preetha, R., M. Jasmine Pemeena Priyadarsini, and J. S. Nisha. "Automated Brain Tumor Detection from Magnetic Resonance Images Using Fine-Tuned EfficientNet-B4 Convolutional Neural Network." *IEEE Access* (2024).

26. Wang, Yaoli, Yaojun Deng, Yuanjin Zheng, Pratik Chattopadhyay, and Lipo Wang. "Vision Transformers for Image Classification: A Comparative Survey." *Technologies* 13, no. 1 (2025): 32

Comment 2: What pretraining weights were used for the AI models?

**Response:** We appreciate this request for clarification. As stated in the manuscript METHODS Section, Sub- Section 2.2.1 lines 209-210:

“Initialization of the model weights was from a pre-trained state on the ImageNet dataset, leveraging transfer learning to enhance initial performance and accelerate convergence.”

Comment 3: The authors should perform the analysis for the state of the art methods including vision transformers as well.

**Response:** We appreciate the reviewer’s suggestion regarding Vision Transformers (ViTs) as a state-of-the-art approach in medical imaging. However, the primary objective of this study is not to benchmark different deep learning architectures but rather to explore how demographic biases in training data affect the performance of AI models in musculoskeletal ultrasound. Given that demographic biases arise from imbalances in data representation rather than specific model architectures, the effect of bias on ViTs is expected to be similar to that observed in CNN-based models.

We selected CNN-based models, specifically EfficientNet-B4, due to their efficiency, widespread adoption in medical imaging applications, and suitability for datasets of moderate size. While ViTs have demonstrated superior performance in large-scale computer vision tasks, their application in clinical AI is still evolving, and they typically require significantly larger datasets and computational resources to outperform CNNs.

Nevertheless, we acknowledge the potential of ViTs in future medical AI applications. To reflect this, we have added the following paragraphs in INTRODUCTION Section lines 127-133:

“While ViTs introduce self-attention mechanisms that enhance feature extraction, the effect of dataset bias on ViTs is expected to be similar to that on CNNs—model fairness and generalization are primarily dictated by the composition and diversity of the training data, rather than architecture choice. CNNs, particularly architectures like EfficientNet, remain widely used in real-world medical settings because of their computational efficiency, established performance, and ability to generalize well on moderate-sized datasets (25,26).”

and DISCUSSION Section, Sub-Section 4-1, lines 491-496:

“Bias in AI models is primarily driven by dataset composition rather than architecture choice. Regardless of whether CNNs or ViTs are used, training data diversity and representativeness remain the most critical factors in ensuring fairness and mitigating bias in diagnostic models. Future research could investigate whether ViTs amplify or mitigate demographic biases in musculoskeletal ultrasound classification by leveraging their attention-based feature extraction.”

25.Preetha, R., M. Jasmine Pemeena Priyadarsini, and J. S. Nisha. "Automated Brain Tumor Detection from Magnetic Resonance Images Using Fine-Tuned EfficientNet-B4 Convolutional Neural Network." *IEEE Access* (2024).

26. Wang, Yaoli, Yaojun Deng, Yuanjin Zheng, Pratik Chattopadhyay, and Lipo Wang. "Vision Transformers for Image Classification: A Comparative Survey." *Technologies* 13, no. 1 (2025): 32

Comment 4: The methods based on self-supervised learning should also be considered. How will the bias be for the models which are fine-tuned when initialized with SSL weights such as DINOv2.

**Response:** We appreciate the reviewer's suggestion regarding self-supervised learning (SSL) methods such as DINOv2. SSL-based models have shown potential in learning robust and transferable representations, particularly in medical imaging applications where labeled data is limited. These approaches may help mitigate certain biases by leveraging diverse, unlabeled datasets to pretrain models before fine-tuning on specific tasks.

In our study, the primary objective was to investigate the impact of demographic biases in training data on diagnostic accuracy, rather than optimizing pretraining strategies. We selected CNN-based models trained in a supervised manner to ensure compatibility with widely adopted clinical AI frameworks. While SSL approaches such as DINOv2 and SimCLR offer promising avenues for mitigating bias, their effectiveness in musculoskeletal ultrasound classification remains an open question. We acknowledge this as a limitation and have now included a discussion in the revised manuscript, suggesting future work to explore SSL-based pretraining and assess its influence on demographic fairness in AI models. We have added the following discussion in DISCUSION Section, Sub-Section 4-1, lines 497-505:

"Self-supervised learning (SSL) methods, such as DINOv2 and SimCLR, have demonstrated strong generalization capabilities in medical imaging, particularly in scenarios where labeled data is limited. These approaches leverage large-scale, unlabeled datasets for pretraining, potentially enabling models to learn robust feature representations that generalize across demographic groups. Incorporating SSL-based pretraining could influence how models learn from imbalanced demographic distributions and mitigate bias in musculoskeletal ultrasound classification. Future work should evaluate SSL-based architectures in this context, comparing their ability to reduce bias against conventional supervised learning approaches."

Comment 5: Comparison to foundation models is also missing. The authors should analyze how the foundation models in this field perform in terms of sex bias. Both in zero-shot and fine-tuned with their training data scenario.

We appreciate the reviewer’s suggestion regarding the inclusion of foundation models for comparative analysis. In this study, we aimed to assess the impact of demographic biases on diagnostic performance using CNN-based models, which remain widely used in clinical AI applications due to their efficiency, interpretability, and feasibility for deployment in resource-constrained medical environments.

We acknowledge that foundation models, such as SAM and MedCLIP, have demonstrated strong generalization capabilities across diverse medical imaging tasks. Their large-scale pretraining allows them to perform well in zero-shot settings and adapt effectively through fine-tuning. However, the evaluation of foundation models in terms of demographic bias, particularly in musculoskeletal ultrasound classification, remains an open research question. Given that these models are often trained on broad and heterogeneous datasets, their ability to mitigate bias in domain-specific applications like ours warrants further exploration.

To address this, we have now included a discussion on foundation models in the manuscript’s Discussion section, acknowledging their potential role in bias mitigation and proposing their evaluation as a direction for future work. We have added the following in the DISCUSION Section, Sub-Section 4-1, lines 506-516:

“The rapid advancement of foundation models, such as SAM and MedCLIP, has introduced new opportunities for improving generalization and fairness in medical imaging. These models, pretrained on large-scale multimodal datasets, have demonstrated robust performance in zero-shot and fine-tuned settings across various medical tasks. However, their effectiveness in mitigating demographic bias in musculoskeletal ultrasound classification remains largely unexplored. Future research should investigate how foundation models perform in terms of sex bias, both in zero-shot inference and when fine-tuned on domain-specific ultrasound data. While our current study focuses on CNN-based classifiers due to their clinical relevance and computational feasibility, extending this analysis to foundation models could provide further insights into mitigating bias in AI-driven diagnostics.”

Comment 6: The statistical analysis should be revisited. The authors currently base everything on the n=5 of five folds of validation. This is not a very representative statistical analysis. The authors should set a held-out test set fixed across all experiments and perform strictly paired analyses. To get the statistical measures (mean +- SD, as well as p-values for the comparisons) the authors may use bootstrapping.

**Response**: We thank the reviewer for this suggestion. In response, we have implemented a paired bootstrapping analysis on a fixed, balanced test set to rigorously assess the performance differences among our three training scenarios:

We have added the following paragraph in the METHODS Section, Sub-Section 2.2.2- lines 227-238:

“To provide a more robust statistical evaluation, we employed a paired bootstrapping analysis on a fixed, balanced test set (29). The test set remained constant across all experimental conditions, ensuring equal representation of male and female images. For each pairwise comparison, we performed 5,000 iterations of bootstrapping, where we resampled the test set with replacement and computed the accuracy difference between models at each iteration. From these resampled distributions, we calculated the mean accuracy difference, standard deviation, and 95% confidence intervals, along with p-values to assess statistical significance. This method ensures a statistically rigorous evaluation of how demographic bias in training data affects model performance across three training scenarios: (1) Male-Only, (2) Female-Only, and (3) Balanced Training. This approach ensures a statistically sound assessment of demographic bias effects on model performance.”

Additionally, in the RESULTS Section lines 325-348:

“Paired bootstrapping analysis revealed significant performance differences based on training data composition. The Male-Only model consistently underperformed compared to both the Female-Only and Balanced models, indicating that training exclusively on male data led to reduced diagnostic accuracy. Our statistical findings, based on 5,000 iterations, are as follows:

Male-Only vs. Female-Only:

Mean Difference (Male - Female): -3.47 percentage points

Standard Deviation of Difference: 1.13 percentage points

95% Confidence Interval: [-5.65%, -1.41%]

p-value: 0.0030

Male-Only vs. Balanced:

Mean Difference (Male - Balanced): -3.48 percentage points

Standard Deviation of Difference: 1.07 percentage points

95% Confidence Interval: [-5.54%, -1.41%]

p-value: 0.0020

Female-Only vs. Balanced:

Mean Difference (Female - Balanced): 0.01 percentage points

Standard Deviation of Difference: 0.97 percentage points

95% Confidence Interval: [-1.85%, 1.85%]

p-value: 0.5180

The Male-Only model showed a statistically significant reduction in accuracy compared to both the Female-Only and Balanced models (p < 0.005). In contrast, the Female-Only and Balanced models performed similarly (p = 0.5180), suggesting that female-trained models generalized as well as balanced models.”

Finally, in the DISCUSION Section lines 412-427:

“The paired bootstrapping results confirm that demographic biases in training data significantly affect model performance. Our analysis demonstrates that models trained solely on male data perform consistently worse than those trained on female-only or balanced datasets. This suggests that male ultrasound images alone may not provide sufficient variability for robust generalization, highlighting the need for diverse datasets to achieve equitable AI performance in musculoskeletal ultrasound.

Moreover, the significant performance gap between Male-Only and Female-Only models (p = 0.0030) suggests that sex differences in musculoskeletal anatomy influence AI-based classification. Given the lack of significant performance differences between the Female-Only and Balanced models (p = 0.5180), these findings further indicate that models trained on female-only data generalize more effectively.

These results reinforce the necessity of balanced, demographically diverse training datasets to ensure fair and reliable diagnostic performance across patient populations. Future studies should investigate whether additional factors, such as age and anatomical variations, further impact model performance, as well as explore strategies like domain adaptation or augmentation techniques to mitigate dataset imbalances.”

29.Efron, Bradley, and Robert J. Tibshirani. *An introduction to the bootstrap*. Chapman and Hall/CRC, 1994.

Comment 7: The literature review is not complete and many of the important references related to this study are missing.

**Response**: Thank you for pointing this out. We have added the following paragraphs in INTRODUCTION Section, lines 106-117:

“A growing body of clinical research highlights significant sex differences in musculoskeletal anatomy and osteoarthritis (OA). Systematic reviews and clinical studies have documented that women often experience a higher prevalence of OA, along with distinct morphometric differences in joint structures, compared to men (23). These differences are attributed to a complex interplay of hormonal, genetic, and biomechanical factors, which may render female joints more vulnerable to degenerative changes. Moreover, emerging evidence suggests that AI models trained on unbalanced datasets might inadvertently reinforce these biases, leading to disparate diagnostic outcomes across sexes (24). Overall, these studies not only provide a critical clinical context for understanding the impact of sex on OA but also highlight the necessity for balanced, demographically representative training datasets to ensure equitable performance in AI-driven diagnostics.

And lines 127-133:

“While ViTs introduce self-attention mechanisms that enhance feature extraction, the effect of dataset bias on ViTs is expected to be similar to that on CNNs—model fairness and generalization are primarily dictated by the composition and diversity of the training data, rather than architecture choice. CNNs, particularly architectures like EfficientNet, remain widely used in real-world medical settings because of their computational efficiency, established performance, and ability to generalize well on moderate-sized datasets (25,26).”

And in DISCUSSION Section lines 491-496:

“Bias in AI models is primarily driven by dataset composition rather than architecture choice. Regardless of whether CNNs or ViTs are used, training data diversity and representativeness remain the most critical factors in ensuring fairness and mitigating bias in diagnostic models. Future research could investigate whether ViTs amplify or mitigate demographic biases in musculoskeletal ultrasound classification by leveraging their attention-based feature extraction.”

23. Cross JL, Choma MA, Onofrey JA. Bias in medical AI: Implications for clinical decision-making. PLOS Digital Health. 2024;3(11):e0000651.

24. Latif E, Zhai X, Liu L. Ai gender bias, disparities, and fairness: Does training data matter?. arXiv preprint arXiv:2312.10833. 2023

25. Preetha R, Priyadarsini MJP, Nisha JS. Automated Brain Tumor Detection from Magnetic Resonance Images Using Fine-Tuned EfficientNet-B4 Convolutional Neural Network. IEEE Access. 2024.

26. Wang Y, Deng Y, Zheng Y, Chattopadhyay P, Wang L. Vision Transformers for Image Classification: A Comparative Survey. Technologies. 2025;13(1):32.

Comment 8: More figures could be used in this manuscript for better and easier explanation of the contents for the reader. Moreover, figure 1 is not very easily readable.

**Response**: We thank the reviewer for their valuable feedback. We have revised Figure 2 (Fig 1 in the first submission), now it is easier to read. Additionally, we have added figure (Fig. 1) to better illustrate the approach of the paper in a flowchart format. Furthermore, we have included (Figure 3) showcasing Grad-CAM visualizations for the sex classification model, providing clearer insights into the regions of the ultrasound images the model focuses on.

We have added the following paragraph in INTRODUCTION Section, line 134-143

“Figure 1 provides a comprehensive overview of the study workflow, outlining the key stages from data preparation to model evaluation and bias analysis. The pipeline begins with dataset preparation, including expert screening and annotation, followed by data partitioning and cross-validation. The model training phase employs EfficientNet-B4 across three training scenarios (Male-Only, Female-Only, and Balanced), incorporating data augmentation strategies to enhance generalizability. Finally, model performance is evaluated through multiple metrics, including accuracy, sensitivity, specificity, and AUC, with additional bias analysis using Grad-CAM visualization to interpret model decisions across different training conditions. This structured approach ensures a thorough investigation of demographic biases in AI-driven musculoskeletal ultrasound diagnosis.”

Figure 1. Overview of the study workflow for AI-driven musculoskeletal ultrasound analysis. The pipeline consists of three main stages: (1) Data Preparation, including expert screening, dataset composition, and partitioning; (2) Model Training, utilizing EfficientNet-B4 with different training scenarios and data augmentation strategies; and (3) Evaluation & Bias Analysis, which involves performance assessment, Grad-CAM visualization, and bias comparison across training conditions.

Figure 3. Grad-CAM visualizations of the sex-classification model applied to knee joint ultrasound images. The heatmaps highlight the regions that contribute most to the model's predictions, illustrating potential sex-specific imaging features. These visualizations provide insights into the model’s decision-making process and help assess possible sources of bias in classification.

Comment 9: How can we see the source code of this work for reproduction?

**Response:** We thank the reviewer for this comment. To ensure reproducibility and transparency, we acknowledge the importance of providing access to the source code. While data cannot be publicly shared due to ethical and privacy considerations, we have made the core implementation of our model, preprocessing pipeline, and evaluation methodology available in a public repository.

We have added the following: in METHODS Section, Sub-Section 2.2.2, lines 205-208:

“To ensure reproducibility and transparency, we acknowledge the importance of providing access to the source code. While data cannot be publicly shared due to ethical and privacy considerations, we have made the core implementation of our model, preprocessing pipeline, and evaluation methodology available in https://github.com/Noushh/PLOS-One-paper.”

To sum up, the presented results cannot be accepted as general findings in this field yet. The authors only used a specific type of architecture (CNN) and only a specific small dataset, and only one paradigm of methodology, no validation of the results on public data, and without proper statical significance analyses.

Reviewer #2: Strengths:

-This paper is the first to explore the problem of sex bias on mskus

-The study highlights important aspects that need to be further studied

-The paper is well-written and well-organized

Weaknesses:

-The main claim is not supported by the results

-Missing a statistical significance test

-Missing details for reproducibility

-Lack of a discussion on the implications and possible solutions

Detailed comment:

The main claim of the paper is to highlight the demographic bias on diagnostic accuracy, suggesting the need for more diverse training data. The experiments carried out in the paper are not sufficient to support such a claim. The difference in the results might be solely due to the different training and validation sets, that change for each subset. With such a claim I expected to have the model trained on the male subgroup to outperform the one trained on the female for the male test data, and so on, while results show that in some cases the best performing model of a subgroup is the one trained on the other subgroup. I expected that the one trained on both was outperforming the others on all the metrics while it happens for only some of them, thus invalidating the claim that it has increased generalizability.

To support such claims a statistical significance test should be performed between the various groups to ensure that the difference in results is relevant.

**Response**: We thank the reviewer for this suggestion. In response, we have implemented a paired bootstrapping analysis on a fixed, balanced test set to rigorously assess the performance differences among our three training scenarios:

We have added the following paragraph in the METHODS Section, Sub-Section 2.2.2- line 227-238:

“To provide a more robust statistical evaluation, we employed a paired bootstrapping analysis on a fixed, balanced test set (29). The test set remained constant across all experimental conditions, ensuring equal representation of male and female images. For each pairwise comparison, we performed 5,000 iterations of bootstrapping, where we resampled the test set with replacement and computed the accuracy difference between models at each iteration.

From these resampled distributions, we calculated the mean accuracy difference, standard deviation, and 95% confidence intervals, along with p-values to assess statistical significance. This method ensures a statistically rigorous evaluation of how demographic bias in training data affects model performance across three training scenarios:

Male-Only Training

Female-Only Training

Balanced Training (Male and Female)

Additionally, in the RESULTS Section line 325-348:

“Paired bootstrapping analysis revealed significant performance differences based on training data composition. The Male-Only model consistently underperformed compared to both the Female-Only and Balanced models, indicating that training exclusively on male data led to reduced diagnostic accuracy. Our statistical findings, based on 5,000 iterations, are as follows:

Male-Only vs. Female-Only:

Mean Difference (Male - Female): -3.47 percentage points

Standard Deviation of Difference: 1.13 percentage points

95% Confidence Interval: [-5.65%, -1.41%]

p-value: 0.0030

Male-Only vs. Balanced:

Mean Difference (Male - Balanced): -3.48 percentage points

Standard Deviation of Difference: 1.07 percentage points

95% Confidence Interval: [-5.54%, -1.41%]

p-value: 0.0020

Female-Only vs. Balanced:

Mean Difference (Female - Balanced): 0.01 percentage points

Standard Deviation of Difference: 0.97 percentage points

95% Confidence Interval: [-1.85%, 1.85%]

p-value: 0.5180

The Male-Only model showed a statistically significant reduction in accuracy compared to both the Female-Only and Balanced models (p < 0.005). In contrast, the Female-Only and Balanced models performed similarly (p = 0.5180), suggesting that female-trained models generalized as well as balanced models.”

Finally, in the DISCUSION Section lines 412-427:

“The paired bootstrapping results confirm that demographic biases in training data significantly affect model performance. Our analysis demonstrates that models trained solely on male data perform consistently worse than those trained on female-only or balanced datasets. This suggests that male ultrasound images alone may not provide sufficient variability for robust generalization, highlighting the need for diverse datasets to achieve equitable AI performance in musculoskeletal ultrasound.

Moreover, the significant performance gap between Male-Only and Female-Only models (p = 0.0030) suggests that sex differences in musculoskeletal anatomy influence AI-based classification. Given the lack of significant performance differences between the Female-Only and Balanced models (p = 0.5180), these findings further indicate that models trained on female-only data generalize more effectively.

These results reinforce the necessity of balanced, demographically diverse training datasets to ensure fair and reliable diagnostic performance across patient populations. Future studies should investigate whether additional factors, such as age and anatomical variations, further impact model performance, as well as explore strategies like domain adaptation or augmentation techniques to mitigate dataset imbalances.”

29. Efron, B., & Tibshirani, R. (1993). "An Introduction to the Bootstrap." Chapman & Hall/CRC.

.”

As the second point, I find the work missing some methodological details to ensure reproducibility, as I understand that the data cannot be shared, the methodological choices should be accurately reported. As an example there is no clear description of how the 5000 images were selected from the bigger cohort, and how the 80/20 split was performed, is it stratified? Is it patient-based? Also pre-processing and training procedures need to be better described: data augmentation is relevant and should be explained accurately, same for the training parameters as the scheduler, which policy was used?

**Response:** We thank the reviewer for this comment. To ensure reproducibility and transparency, we acknowledge the importance of providing access to the source code. While data cannot be publicly shared due to ethical and privacy considerations, we have made the core implementation of our model, preprocessing pipeline, and evaluation methodology available in a public repository.

We have added the following: in METHODS Section, Sub-Section 2.2.2, lines 205-208:

“To ensure reproducibility and transparency, we acknowledge the importance of providing access to the source code. While data cannot be publicly shared due to ethical and privacy considerations, we have made the core implementation of our model, preprocessing pipeline, and evaluation methodology available in https://github.com/Noushh/PLOS-One-paper.”

Minor comments:

On 3.3 it would be interesting to see the grad-cam of the sex-classification model to better understand the features that are used by the model to make the decisions. This could be important to understand the implication and the possible bias [1]. Also, just citing the “robustness mirroring the results” is not descriptive enough: report the exact results.

**Response:** Thanks for your valuable suggestion. We have added the following in the RESULTS Sectio,, Sub-Section 3.3, line 374-383:

“To further interpret the decision-making process of the sex-classification model, we utilized Grad-CAM to visualize the regions of interest in ultrasound images that contributed most significantly to the model’s predictions. **Figure 3** illustrates heatmaps generated for both male and female classifications. The model primarily focuses on tissue structures surrounding the knee joint, suggesting that differences in soft tissue characteristics or imaging artifacts might be leveraged for classification. However, some heatmaps reveal a reliance on non-anatomical regions, which raises potential concerns regarding bias in the learned representations. These findings highlight the importance of interpretability methods in medical AI to ensure that models base their decisions on clinically relevant features rather than spurious correlations.”

Figure 3. Grad-CAM visualizations of the sex-classification model applied to knee joint ultrasound images. The heatmaps highlight the regions that contribute most to the model's predictions, illustrating potential sex-specific imaging features. These visualizations provide insights into the model’s decision-making process and help assess possible sources of bias in classification.

In a work where the methodological contribution is minor, a more in-depth analysis of the consequences of a biased model and the possible solution is needed to give strength to the paper.

**Response:** We thank the reviewer for this comment. To address the comment, we have revise the DISCUSION Section, lines 392-396 as follows:

“Our study highlights the presence of sex bias in AI-driven musculoskeletal ultrasound diagnosis, revealing that models trained on male-only data perform worse than those trained on female-only or balanced datasets. These findings emphasize the need for careful dataset curation to mitigate performance disparities and improve clinical applicability. Given the widespread integration of AI in medical imaging, it is critical to address demographic biases that could disproportionately impact certain patient groups.”

And lines 491-496:

“Bias in AI models is primarily driven by dataset composition rather than architecture choice. Regardless of whether CNNs or ViTs are used, training data diversity and representativeness remain the most critical factors in ensuring fairness and mitigating bias in diagnostic models. Future research could investigate whether ViTs amplify or mitigate demographic biases in musculoskeletal ultrasound classification by leveraging their attention-based feature extraction.”

And lines 497-516:

“Self-supervised learning (SSL) methods, such as DINOv2 and SimCLR, have demonstrated strong generalization capabilities in medical imaging, particularly in scenarios where labeled data is limited. These approaches leverage large-scale, unlabeled datasets for pretraining, potentially enabling models to learn robust feature representations that generalize across demographic groups. Incorporating SSL-based pretraining could influence how models learn from imbalanced demographic distributions and mitigate bias in musculoskeletal ultrasound classification. Future work should evaluate SSL-based architectures in this context, comparing their ability to reduce bias against conventional supervised learning approaches.

The rapid advancement of foundation models, such as SAM and MedCLIP, has introduced new opportunities for improving generalization and fairness in medical imaging. These models, pretrained on large-scale multimodal datasets, have demonstrated robust performance in zero-shot and fine-tuned settings across various medical tasks. However, their effectiveness in mitigating demographic bias in musculoskeletal ultrasound classification remains largely unexplored. Future research should investigate how foundation models perform in terms of sex bias, both in zero-shot inference and when fine-tuned on domain-specific ultrasound data. While our current study focuses on CNN-based classifiers due to their clinical relevance and computational feasibility, extending this analysis to foundation models could provide further insights into mitigating bias in AI-driven diagnostics.”

In 4.1 The authors talk about further experiments with significant improvement, what are these experiments? No reference to them is reported, why not show directly these results? Either include sufficient information or remove this from the paper.

**Response:** We appreciate the reviewer’s concern. We explicitly state that the mentioned “further experiments” refer to ongoing pilot studies involving a larger dataset collected from additional clinical sites. These experiments are intended to explore the generalizability of our findings across different patient populations and imaging conditions. However, these pilot experiments are not yet fully validated, and the results are still under review. To prevent premature conclusions, we have opted not to include these data in the main manuscript.

To address this concern, we have revised Section 4.1 lines 466-478 as follows:

“While the initial recess distension detection accuracy of approximately 85% may be considered below optimal clinical standards, our analysis underscores the importance of dataset size and diversity in improving AI performance. Expanding the dataset size in preliminary investigations indicated performance improvements, with accuracy increasing to 92%. However, we do not report these additional results in detail, as they require further validation. Interestingly, the performance of the patient sex classification model declined when trained with a larger dataset, suggesting that increased variability in data may introduce additional challenges. These findings highlight the dual impact of dataset size: while larger and more representative datasets help mitigate overfitting and improve generalizability across patient populations, they may also introduce complexities that require more sophisticated modeling approaches. This underscores the need for careful dataset curation to ensure AI models are both accurate and unbiased in clinical applications.”

[1] Li, David, et al. "Deep learning prediction of sex on chest radiographs: a potential contributor to biased algorithms." Emergency Radiology 29.2 (2022)

Reviewer #3: Introduction:

1. I think the study should provide some information how the AI works in assessing diagnosis of synovial recess distension (Like the general algorithm). This information in introduction will be essential to determine or show the gap research that the author want to deliver.

**Response**: We appreciate the reviewer’s suggestion and have revised the Introduction to provide a more detailed explanation of how AI models assess synovial recess distension. Specifically, we have added a paragraph describing the general workflow of AI-based classification in musculoskeletal ultrasound. This new addition outlines the preprocessing steps, feature extraction using deep learning models, and classification mechanisms used to distinguish between normal and distended synovial recesses. Furthermore, we highlight the existing challenges associated with demographic biases in training data, which is the central research gap this study aims to address. We have added the following paragraph in INTRODUCTION Section, lines 72-86

“CNNs have shown promise in automating the diagnosis of musculoskeletal conditions by analyzing ultrasound images. The general pipeline for AI-assisted diagnosis of synovial recess distension follows a structured approach: (1) preprocessing of ultrasound images to enhance relevant anatomical structures, (2) feature extraction using deep learning models, and (3) classification based on learned representations distinguishing between normal and distended synovial recesses. Previous research has demonstrated that CNNs can effectively identify fluid accumulation in synovial recesses, aiding in the early detection of inflammatory conditions such as arthritis. However, these models are susceptible to dataset biases, particularly those related to patient demographics such as sex, which can impact diagnostic accuracy and generalizability. Despite advancements in AI for musculoskeletal ultrasound, there remains a significant gap in understanding how demographic factors influence model performance. This study aims to address this gap by systematically evaluating the effects of training data composition on AI-driven classification of synovial recess distension.”

Method

1. In Image Review and Validation, the author mentioned that a preliminary screening process conducted by an expert sonographer with extensive experience (32 years), is only one expert ask for this screening? Why does the author consider more than one expert to avoid any bias operator?

**Response:** We acknowledge the reviewer’s concern regarding potential operator bias in the image screening process. The preliminary image review was conducted by a highly experienced sonographer with 32 years of expertise in musculoskeletal ultrasound to ensure consistency in quality assessment and inclusion criteria. To address the reviewer’s comment, we have added the following paragraph in the DISCUSION Section, Sub-Section 4-1lines 460-465:

“A potential limitation of our study is that the preliminary image review was conducted by a single expert sonographer with extensive experience (32 years). While the expert followed a strict protocol to ensure consistency in image selection, the involvement of only one reviewer may introduce a degree of subjectivity. In future studies, a multi-expert validation approach will be implemented to assess inter-rater reliability and mitigate potential operator bias.”

Results

1. The author has delivered the results of the research questions very well. The results are informative. However, in the section 3.3 Model for the Determination of Patient Sex Using Knee Joint Ultrasound. The author did not convey the meaning of Determination of Patient Sex Using Knee Joint Ultrasound is mean. The author should explain more about the meaning of this result.

**Response:** We thank the reviewer for the comment. In this study, we trained a binary classification model to predict patient sex (male vs. female) based solely on knee joint ultrasound images. The goal of this analysis was to investigate whether sex-related anatomical differences in the knee joint could be learned by deep learning models. Evaluating the performance of this classifier allows us to assess the presence and significance of these differences in musculoskeletal ultrasound imaging.

We have added the following paragraph in RESULTS Section, Sub-Section 3.3, lines 367-373:

“The high accuracy of the sex classifier confirms that deep learning models can distinguish sex-related anatomical differences in knee ultrasound images. This finding underscores the influence of patient sex on learned feature representations in AI-driven musculoskeletal diagnosis. If diagnostic models inadvertently rely on these sex-specific features, they may introduce bias, particularly when trained on unbalanced datasets. Understanding these inherent differences is therefore crucial for developing fair and reliable AI-based medical imaging applications.”

Discussion

1. I think this part needs a lot of improvement. Results have been very well delivered but it is unfortunate that they are not discussed deeply enough in the discussion section, including the main research question, which is the sex bias in diagnostic. I have yet to see a solid and comprehensive discussion between the author's findings and previous literature or other facts. Every finding that has been well presented in the results also does not appear to be reviewed in the discussion part.

**Response:** We thank the reviewer for their comment. We have added the following paragraph in the DISCUSSION Section, line 392-397:

“Our study highlights the presence of sex bias in AI-driven musculoskeletal ultrasound diagnosis, revealing that models trained on male-only data perform worse than those trained on female-only or balanced datasets. These findings emphasize the need for careful dataset curation to mitigate performance disparities and improve clinical applicability. Given the widespread integration of AI in medical imaging, it is critical to address demographic biases that could disproportionately impact certain patient groups.”

And line 427-435:

“Our results align with previous studies demonstrating demographic biases in AI models trained on imbalanced datasets. For example, (30,31) found that sex-based differences in training data significantly influenced the sensitivity and specificity of diagnostic AI models. Similarly, (32) highlighted that models trained on a single demographic subgroup tend to overfit to their characteristics, reducing generalizability. Our findings extend this work by providing robust statistical evidence through paired bootstrapping analysis, confirming that male-only training yields lower diagnostic performance compared to female-only or balanced training scenarios.”

30. *“Shortcuts” causing bias in radiology artificial intelligence: causes, evaluation, and mitigation.* Banerjee, Imon, et al. s.l.: Journal of the American College of Radiology, 2023.

31. *Gender imbalance in medical imaging datasets produces biased classifiers for computer-aided diagnosis.* Larrazabal, Agostina J., et al. s.l.: Proceedings of the National Academy of Sciences, 2020.

32. *Understanding biases and disparities in radiology AI datasets: a review.* Tripathi, Satvik, et al. s.l. : ournal of the American College of Radiology, 2023 .

2. In addition, in the discussion section, I think the author failed to convey the main message of this research, the recommendations obtained, and research questions that can be developed in the future with similar themes.

**Response:** We thank the reviewer for their comment. We have added the following paragraph in the DISCUSSION Section, lines 392-397:

“Our study highlights the presence of sex bias in AI-driven musculoskeletal ultrasound diagnosis, revealing that models trained on male-only data perform worse than those trained on female-only or balanced datasets. These findings emphasize the need for careful dataset curation to mitigate performance disparities and improve clinical applicability. Given the widespread integration of AI in medical imaging, it is critical to address demographic biases that could disproportionately impact certain patient groups.”

Future studies should investigate whether foundation models trained on large-scale medical datasets exhibit less demographic bias than CNNs. Additionally, domain adaptation techniques and self-supervised learning strategies should be explored as potential methods for improving model generalization across diverse patient populations. Addressing these open questions will be essential for the equitable deployment of AI in medical imaging.

**Response:** We thank the reviewer for this comment. To address the comment, we have revise the DISCUSION Section, lines 392-397 as follows:

“Our study highlights the presence of sex bias in AI-driven musculoskeletal ultrasound diagnosis, revealing that models trained on male-only data perform worse than those trained on female-only or balanced datasets. These findings emphasize the need for careful dataset curation to mitigate performance disparities and improve clinical applicability. Given the widespread integration of AI in medical imaging, it is critical to address demographic biases that could disproportionately impact certain patient groups.”

And the following to the INTRODUCTION Section, lines 127-133:

“While ViTs introduce self-attention mechanisms that enhance feature extraction, the effect of dataset bias on ViTs is expected to be similar to that on CNNs—model fairness and generalization are primarily dictated by the composition and diversity of the training data, rather than architecture choice. CNNs, particularly architectures like EfficientNet, remain widely used in real-world medical settings because of their computational efficiency, established performance, and ability to generalize well on moderate-sized datasets (25,26).”

And the following to the DISCUSSION Section, Sub-Section 4.1, 491-496

“Bias in AI models is primarily driven by dataset composition rather than architecture choice. Regardless of whether CNNs or ViTs are used, training data diversity and representativeness remain the most critical factors in ensuring fairness and mitigating bias in diagnostic models. Future research could investigate whether ViTs amplify or mitigate demographic biases in musculoskeletal ultrasound classification by leveraging their attention-based feature extraction.”

And lines 497-516:

“Self-supervised learning (SSL) methods, such as DINOv2 and SimCLR, have demonstrated strong generalization capabilities in medical imaging, particularly in scenarios where labeled data is limited. These approaches leverage large-scale, unlabeled datasets for pretraining, potentially enabling models to learn robust feature representations that generalize across demographic groups. Incorporating SSL-based pretraining could influence how models learn from imbalanced demographic distributions and mitigate bias in musculoskeletal ultrasound classification. Future work should evaluate SSL-based architectures in this context, comparing their ability to reduce bias against conventional supervised learning approaches.

The rapid advancement of foundation models, such as SAM and MedCLIP, has introduced new opportunities for improving generalization and fairness in medical imaging. These models, pretrained on large-scale multimodal datasets, have demonstrated robust performance in zero-shot and fine-tuned settings across various medical tasks. However, their effectiveness in mitigating demographic bias in musculoskeletal ultrasound classification remains largely unexplored. Future research should investigate how foundation models perform in terms of sex bias, both in zero-shot inference and when fine-tuned on domain-specific ultrasound data. While our current study focuses on CNN-based classifiers due to their clinical relevance and computational feasibility, extending this analysis to foundation models could provide further insights into mitigating bias in AI-driven diagnostics.”

25.Preetha, R., M. Jasmine Pemeena Priyadarsini, and J. S. Nisha. "Automated Brain Tumor Detection from Magnetic Resonance Images Using Fine-Tuned EfficientNet-B4 Convolutional Neural Network." *IEEE Access* (2024).

26. Wang, Yaoli, Yaojun Deng, Yuanjin Zheng, Pratik Chattopadhyay, and Lipo Wang. "Vision Transformers for Image Classification: A Comparative Survey." *Technologies* 13, no. 1 (2025): 32

6. PLOS authors have the option to publish the peer review history of their article (what does this mean?). If published, this will include your full peer review and any attached files.

If you choose “no”, your identity will remain anonymous but your review may still be made public.

Do you want your identity to be public for this peer review? For information about this choice, including consent withdrawal, please see our Privacy Policy.

Reviewer #1: No

Reviewer #2: No

Reviewer #3: No
