# Supplementary material for: Evaluating the impact of sex bias on AI models in musculoskeletal ultrasound of joint recess distension
Source: PLoS One. 2025 Nov 12;20(11):e0332716. doi: 10.1371/journal.pone.0332716 (PMC12611148; doi:10.1371/journal.pone.0332716)
Supplement: Attachment — Submitted filename: Response_to_Reviewers_auresp_2.docx [file pone.0332716.s010.docx]

We thank all the reviewers for their constructive comments, and the Associate Editor for handling the review of this paper. Please find the point-by-point responses to all the comments in blue font. The modifications to the original manuscript are also marked in blue font.

Review Comments to the Author

Reviewer #1:

I thank the authors for revising the manuscript and for their detailed responses. While several of the concerns have been addressed, a few key points remain only partially addressed in the revision. I outline them below.

Comment 1:

While the authors added text acknowledging the role of Vision Transformers, they did not revise the original overstated sentence that describes CNNs as “particularly” revolutionizing the field. This phrasing is misleading and remains uncorrected. I recommend the authors revise this sentence to avoid presenting CNNs as the state-of-the-art. Adding background on ViTs is helpful, but this change should also be reflected in the way the introduction frames CNNs.

**Response:** We thank the reviewer for their feedback. We have revised the sentence in the Introduction, lines 73-74 as follows:

“The integration of artificial intelligence (AI), including convolutional neural networks (CNNs), has significantly advanced early diagnosis practices in medical imaging (8–10).”

Comment 2:
The authors provide a reasonable argument about scope and resources, and they have expanded their discussion to acknowledge the role of ViTs. However, the limitation remains under-communicated to the reader. The authors frame this only as “future work” in the discussion, but it is a major limitation that constrains the generalizability of the findings. This should be mentioned clearly as a limitation in the Abstract and Conclusion, not just in the body of the Discussion.

**Response:** We thank you the reviewer for their suggestion. We have revised both the Abstract and Conclusion to communicate this limitation. We have added the following sentence in the Abstract, lines 53-56:

“These findings are based on a supervised CNN model; evaluating whether they extend to other architectures, such as self-supervised learning (SSL) methods, foundation models, and Vision Transformers (ViTs), remains a direction for future research.”

And in the Conclusion, lines 538-539:

“Future work should assess whether or not these limitations persist when using SSL approaches, foundation models, and ViTs.”

Comment 3:

As with ViTs, the authors expand the discussion but do not perform any experiments or properly elevate this to a clear limitation. Again, this needs to be stated explicitly in the Abstract and Conclusion, otherwise readers may mistakenly assume the findings generalize to SSL-pretrained models, which is not supported by the current experiments.

**Response:** Thank you for pointing this out. We have updated both the Abstract and Conclusion to communicate this limitation. We have added the following sentence in the Abstract, lines 53-56:

“These findings are based on a supervised CNN model; evaluating whether they extend to other architectures, such as self-supervised learning (SSL) methods, foundation models, and Vision Transformers (ViTs), remains a direction for future research.”

And in the Conclusion, lines 538-539:

“Future work should assess whether these limitations persist when using SSL approaches, foundation models, and ViTs.”

Comment 4:

The authors appropriately acknowledge this direction as future work but similarly fail to position it as a present limitation in the Abstract or Conclusion. I encourage them to explicitly state that the current findings are limited to CNNs trained with supervised learning and do not evaluate foundation models, which may behave differently.

**Response:** We appreciate the insightful comment. To address it, we have revised both the Abstract and Conclusion to explicitly state this limitation. The following sentence has been added to the Abstract, lines 53 -56:

“These findings are based on a supervised CNN model; evaluating whether they extend to other architectures, such as self-supervised learning (SSL) methods, foundation models, and Vision Transformers (ViTs), remains a direction for future research.”

And in the Conclusion, lines 538-539:

“Future work should assess whether these limitations persist when using SSL approaches, foundation models, and ViTs.

Reviewer #2:

Comment**:**

I thank the author for taking the presented concerns into consideration. I find the revised version significantly more relevant and well-presented. My only remaining comment is that I would recommend removing the paragraph spanning lines 469–479. While I understand the point being raised, this section should focus solely on presenting the results obtained in the current work and discussing insights derived from those results, not from other unspecified findings.

**Response**: We thank the reviewer for their positive feedback and for acknowledging the improvements made in the revised version. In accordance with the reviewer’s suggestion, we have removed the paragraph in the revised manuscript to ensure that the discussion remains focused solely on the results obtained in the current study.
