# Supplementary material for: Evaluating the impact of sex bias on AI models in musculoskeletal ultrasound of joint recess distension
Source: PLoS One. 2025 Nov 12;20(11):e0332716. doi: 10.1371/journal.pone.0332716 (PMC12611148; doi:10.1371/journal.pone.0332716)
Supplement: Attachment — Submitted filename: Response_to_Reviewers_auresp_3.docx [file pone.0332716.s011.docx]

Thank you for the helpful comments. We have revised the manuscript. All changes are highlighted in blue font.

**Editor Comments:**

1. Please ensure that your manuscript meets PLOS ONE's style requirements, including those for file naming. The PLOS ONE style templates can be found at
<https://journals.plos.org/plosone/s/file?id=wjVg/PLOSOne_formatting_sample_main_body.pdf> andhttps://journals.plos.org/plosone/s/file?id=ba62/PLOSOne_formatting_sample_title_authors_affiliations.pdf.

**Response:** Thank you for the guidance. We have revised the manuscript and file names to fully comply with PLOS ONE’s formatting and style requirements.

2. Cite the name in the main text for the name citation, not the number, for example, in line 449 - 451
While revising your submission, please upload your figure files to the Preflight Analysis and Conversion Engine (PACE) digital diagnostic tool, <https://pacev2.apexcovantage.com/>. PACE helps ensure that figures meet PLOS requirements. To use PACE, you must first register as a user. Registration is free. Then, login and navigate to the UPLOAD tab, where you will find detailed instructions on how to use the tool. If you encounter any issues or have any questions when using PACE, please email PLOS at [figures@plos.org](mailto:figures@plos.org). Please note that Supporting Information files do not need this step.

**Response:** Thank you for pointing this out. We have revised the citation on lines 449–451 (now lines 432–434 in the revised manuscript) to include the author’s name in the main text as requested.

Additionally, the figure files have been uploaded and verified using the PACE system in accordance with PLOS ONE's guidelines.
