# Supplementary material for: Evaluating the impact of sex bias on AI models in musculoskeletal ultrasound of joint recess distension
Source: PLoS One. 2025 Nov 12;20(11):e0332716. doi: 10.1371/journal.pone.0332716 (PMC12611148; doi:10.1371/journal.pone.0332716)
Supplement: Attachment — Submitted filename: Response_to_Reviewers_auresp_4.docx [file pone.0332716.s012.docx]

We would like to thank you for your helpful comments. We have carefully revised the manuscript according to the PLOS ONE style requirements and addressed all the points raised. All modifications are visible using Track Changes in the revised manuscript. Additionally, the figures have been prepared and formatted based on the Preflight Analysis and Conversion Engine (PACE) digital diagnostic requirements.

**Editor Comments:**

1. Please ensure that your manuscript meets PLOS ONE's style requirements, including those for file naming. The PLOS ONE style templates can be found at
<https://journals.plos.org/plosone/s/file?id=wjVg/PLOSOne_formatting_sample_main_body.pdf> and
<https://journals.plos.org/plosone/s/file?id=ba62/PLOSOne_formatting_sample_title_authors_affiliations.pdf>

Thank you for the comment. We have carefully revised the manuscript to align with the PLOS ONE style requirements, including file naming conventions, as outlined in the provided templates.

2. Check again the typos in all parts of the manuscript. For instance, in line 297, it is unclear which figure is cited.

We appreciate your attention to this point. We have thoroughly checked the manuscript for typos and clarified the figure reference. The sentence now clearly reads (line 299):

“Fig 2 shows a visual representation of how bias influences the model's decision-making process.”
3. The reference style is not suited to the journal style. You may check again our guideline: <https://journals.plos.org/plosone/s/submission-guidelines#loc-references>

Thank you for pointing this out. The reference list has been revised to fully comply with the PLOS ONE reference style guidelines.
